# A single-domain antibody for the detection of pathological Tau protein in the early stages of oligomerization

**DOI:** 10.1186/s12967-024-04987-1

**Published:** 2024-02-16

**Authors:** Nicolas De Leiris, Pascale Perret, Charlotte Lombardi, Bülent Gözel, Sabine Chierici, Philippe Millet, Marlène Debiossat, Sandrine Bacot, Benjamin B. Tournier, Patrick Chames, Jean-Luc Lenormand, Catherine Ghezzi, Daniel Fagret, Marcelle Moulin

**Affiliations:** 1 University Grenoble Alpes, Clinique Universitaire de Médecine Nucléaire, INSERM, Centre Hospitalier Universitaire Grenoble Alpes, LRB, CS 10217, 38043 Grenoble CEDEX 9, France; 2 University Grenoble Alpes, INSERM, LRB, 38000 Grenoble, France; 3 University Grenoble Alpes, CNRS, DCM, 38000 Grenoble, France; 4 Division of Adult Psychiatry, Department of Psychiatry, Geneva University Hospitals, Geneva, Switzerland; 5 Aix Marseille University, CNRS, INSERM, Institut Paoli-Calmettes, CRCM, Marseille, France; 6 University Grenoble Alpes, CNRS, TIMC, 38000 Grenoble, France

**Keywords:** Alzheimer’s disease, Tau protein, Oligomers, BBB, Tc-99m, Biomarker, SPECT, sdAb

## Abstract

**Background:**

Soluble oligomeric forms of Tau protein have emerged as crucial players in the propagation of Tau pathology in Alzheimer’s disease (AD). Our objective is to introduce a single-domain antibody (sdAb) named 2C5 as a novel radiotracer for the efficient detection and longitudinal monitoring of oligomeric Tau species in the human brain.

**Methods:**

The development and production of 2C5 involved llama immunization with the largest human Tau isoform oligomers of different maturation states. Subsequently, 2C5 underwent comprehensive in vitro characterization for affinity and specificity via Enzyme-Linked Immunosorbent Assay and immunohistochemistry on human brain slices. Technetium-99m was employed to radiolabel 2C5, followed by its administration to healthy mice for biodistribution analysis.

**Results:**

2C5 exhibited robust binding affinity towards Tau oligomers (Kd = 6.280 nM ± 0.557) and to Tau fibers (Kd = 5.024 nM ± 0.453), with relatively weaker binding observed for native Tau protein (Kd = 1791 nM ± 8.714) and amyloid peptide (Kd > 10,000 nM). Remarkably, this SdAb facilitated immuno-histological labeling of pathological forms of Tau in neurons and neuritic plaques, yielding a high-contrast outcome in AD patients, closely mirroring the performance of reference antibodies AT8 and T22. Furthermore, 2C5 SdAb was successfully radiolabeled with 99mTc, preserving stability for up to 6 h post-radiolabeling (radiochemical purity > 93%). However, following intravenous injection into healthy mice, the predominant uptake occurred in kidneys, amounting to 115.32 ± 3.67, 97.70 ± 43.14 and 168.20 ± 34.52% of injected dose per gram (% ID/g) at 5, 10 and 45 min respectively. Conversely, brain uptake remained minimal at all measured time points, registering at 0.17 ± 0.03, 0.12 ± 0.07 and 0.02 ± 0.01% ID/g at 5, 10 and 45 min post-injection respectively.

**Conclusion:**

2C5 demonstrates excellent affinity and specificity for pathological Tau oligomers, particularly in their early stages of oligomerization. However, the current limitation of insufficient blood–brain barrier penetration necessitates further modifications before considering its application in nuclear medicine imaging for humans.

## Introduction

Alzheimer Disease (AD) is one of the major causes of health decline and dependence among the elderly population, affecting nearly 2% of the population in industrialized countries [1, 2]. It consists of a continuum, starting from an asymptomatic preclinical stage, and progressing to a clinically identifiable disease characterized by means of cognitive and memory tests [3]. At the histopathological level, AD is a neurodegenerative disorder characterized by the cerebral accumulation of two distinctive lesions with extracellular deposits of β-amyloid peptides (Aβ) forming senile plaques and inclusions of abnormally phosphorylated Tau protein filaments primarily located within neurons leading to the formation of neurofibrillary tangles (NFTs) [4–6]. The mechanisms of interaction between these two characteristic neuropathological lesions are not fully understood and are a source of controversy, although these protein lesions seem to have a synergistic effect [7]. These two proteinopathies develop in different sequences and following a different spatial distribution. However, several studies, particularly imaging studies in AD continuum, have identified a dynamic model in which Aβ lesions appear first and serve to trigger the propagation of Tau lesions from the mesial temporal lobe to the cortical regions, this being associated with the progression of cognitive symptoms [8, 9]. The development of new strategies to achieve an early specific diagnosis through the detection of histopathological molecular lesions in individuals at risk is a major public health issue and is of paramount importance [10]. However, substantial diagnostic uncertainties persist during the early symptomatic phase where it is currently difficult to differentiate AD patients at early-stage from other forms of dementia (such as dementia with Lewy bodies or fronto-temporal dementia) or non-degenerative etiologies.

Research in the field has long been polarized around the Aβ cascade since the main hypothesis was based on that the amyloid deposits were the triggering pathway of the disease [11]. However, it has appeared that they do not constitute a predictive biomarker [12]. Moreover, the first treatments targeting amyloid deposition in clinical trials have proven to be unsuccessful [13]. Over the past few years, we have then been witnessing a decline in research activities around the Aβ peptide, in favor of the Tau lesions. Indeed, while Aβ peptide load reaches an early plateau in the AD course and can be observed in cognitively normal elderly, longitudinal clinicopathologic and neuroimaging studies demonstrated that Tau deposition exhibits a stronger correlation with the cognitive decline and neurodegeneration [14–17]. Much more than the NFTs, soluble oligomeric forms of Tau have been shown to be toxic to neurons and involved in the mechanisms of cerebral propagation of Tau pathology from one neuron to another thus illustrating the dynamics of the disease evolution [18–20]. These Tau oligomers essentially contain assemblies of hyperphosphorylated Tau protein as monomers or dimers, and are localized intra-neuronally but also in extracellular regions.

Moreover, various promising active and passive immunotherapeutic strategies, directed against brain pathological forms of Tau and whose impact onto Tau oligomers has been demonstrated in preclinical protocols, are currently being evaluated in clinical trials [21–23]. The development of such disease-modifying therapeutic strategies requires the detection and follow-up of the early neurodegenerative signs of AD, especially the presence of Tau oligomers, in the population at risk of AD. This enables the selection of potential responders to treatment and the assessment of treatment effect. This is all the more interesting as treatments targeting Tau oligomers are currently in development and will require a companion tracer [22].

Thanks to the intravenous injection of a trace amount of radioactive tracers followed by molecular imaging, nuclear medicine is an appropriate tool to provide in vivo evaluation of neuronal damages and pathological protein deposition. Such technique will allow to study both their spatial distribution and quantify their accumulation that which play a leading role in the diagnosis of AD and other dementia conditions. There are already nuclear medicine biomarkers available to study neurodegeneration, such as [^18^F]Fluoro-D-Glucose Positron Emission Tomography (PET), that measures the glucose metabolism, but also radiotracers that can assess specific pathophysiological processes of whom amyloid deposits in PET. In the Tau area, small molecules, targeting β-sheet structures, have already been developed for PET imaging of NFTs, in particular ^18^F-AV-1451 [24] which is currently the only FDA-approved Tau radiotracer [25], although its performances are limited by non-specific binding. However, none of them provide specific access to longitudinal and spatial monitoring of Tau oligomers.

Our aim was to provide a new nuclear imaging ligand to efficiently detect and follow up the extracellular and pathological oligomeric forms of Tau in human brain. In this context, single-domain antibodies (sdAbs) represent a ligand of interest in this context. Indeed, these sdAbs consist of the unique variable domain of naturally occurring heavy-chain antibodies of Camelidae [26–29]. They possess several advantageous structural and functional properties, including high solubility and stability, low immunogenicity in humans and easy to humanize, the ability to recognize epitopes with nanomolar affinity. Moreover, they are more suitable tools than antibodies to cross the blood brain barriers (BBB) due to their smaller molecular weight (~ 15 kDa) and the lack of the Fc Fragment, which prevents export from the brain via the neonatal Fc receptor mediated efflux system present at the BBB. They are also less prone than peptides to proteolytic cleavage. Finally, radiolabeled sdAbs demonstrated a signal to noise ratio propitious for nuclear imaging [30].

In the present study, we have selected, produced and characterized, in vitro and in vivo*,* a sdAb named 2C5 for preclinical imaging of Tau oligomers proposed as an earlier pathological signature of AD.

## Methods

### Single-domain antibody selection

Expression and purification of recombinant Tau protein and production of a Tau fragment containing the R3 repeat region. Prk172-ht40 transformed *E. coli* encoding the longest human Tau isoform (441 amino acids) was a courtesy of M. Goedert, Cambridge, UK [31]. Htau40 production and purification were performed using a modified procedure as described by Novak et al. [32]. In parallel, an acetylated peptide covering the R3 Tau region was synthesized and polymerized to serve as pathological Tau mimes (AcR3).

#### Tau and R3 aggregation

The aggregation of the recombinant Tau protein (110 µM) was facilitated by the presence of heparin (average molecular mass of 8 kDa, Sigma-Aldrich, Cat # H3393; 24 µM) in MOPS buffer (3-(N-morpholino)propanesulfonic acid, Euromedex, Cat # EU0034; 20 mM) containing the reducing agent dithiothreitol (DTT, Sigma-Aldrich, Cat # 11583786001; 1.2 mM) in the presence of phenylmethylsulfonyl fluoride (PMSF, Sigma-Aldrich, Cat # P7626; 5.1 mM). The aggregation was performed at 37 °C for 48 h, 72 h or 16 days [33].

For llama immunization, the AcR3 fibers were prepared from 320 µM AcR3 peptide, 80 µM heparin in the presence of 4 mM DTT and 0.01% sodium azide (NaN_3,_ Sigma-Aldrich, Cat # 822335) in 50 mM phosphate-buffered saline (Dubelcco’s PBS, Dutscher D8662), pH 7.4. After 3 weeks at 37 °C, aliquots of 50 µL were prepared and kept at − 80 °C before using. For ELISA assays and AFM, AcR3 fibrillation was stopped by freezing the samples at − 80 °C after 24 or 48 h.

#### Atomic force microscopy (AFM)

Each AFM sample was prepared by collecting a 3 µL sample from the aggregation step. Each sample was adsorbed onto a freshly cleaved mica surface for 10 min and the surface was washed two times by adding 10 µL of milliQ water (Millipore system, St Quentin en Yvelines, France) and subsequently removing it. AFM images were recorded at randomly selected surface positions in peak force mode using a Dimension Icon (Bruker, Santa Barbara, CA, USA). The cantilevers SCANASIST-Air (Bruker, Santa Barbara, CA, USA) used were triangular and had a force contact of 0.4 N/m and a resonance frequency of 70 kHz at tip scan rates of 1 Hz. AFM processed using the flattening function of the Gwyddion microscope software.

#### Single-domain antibodies generation and production

Tau-oligomers-targeting single-domain antibodies (sdAbs) were generated following established methods described elsewhere [34, 35]. Specifically, a llama was immunized with a mixture consisting of oligomers and fibers of Tau as well as polymers of AcR3 in sterile saline solution, gently mixed with an equal volume of the GERBU Adjuvant F (GERBU Biotechnik GmbH) to make an emulsion and inject subcutaneously (max. 2 mL) in the neck base of the llama. This step was repeated 4 times over a period of 60 days. Total RNA was purified from peripheral blood mononuclear cells (from > 100 mL of blood). The genes coding for the variable domains of the heavy chain-only antibodies (VHHs or sdAbs) were amplified using a reverse transcription polymerase chain reaction (RT-PCR) method and cloned in pHEN1-phoA-6HisGS phagemid, containing a Cmyc tag, a 6xHis tag, and the sequence coding for the protein P3 (allowing the stowage of the phage to the bacterium) and transformed in bacteria *Escherichia coli* TG1. SdAbs were phage-displayed and used in biopanning on immobilized immunogens. Crude bacterial extracts containing soluble sdAbs were used to screen for individual oligomers binders able to recognize Tau pathological forms with a high affinity, and poor affinity for Tau native based on a signal in ELISA. Sequences were obtained (GATC Biotech) to identify monoclonal sdAbs and then only one clone was retained: 2C5. The selected sdAb was produced in BL21 (DE3) *Escherichia coli* and purified, as described previously [36] and later, was produced in large scale in Shuffle T7 Express *Escherichia coli* (New England Biolabs). After transformation, Shuffle T7 express were pre-cultured into a Lysogeny Broth medium with 100 μg/mL of ampicillin (LB-AMP) and were shaken at 180 rpm, 30 °C, overnight. A secondary culture of 1-L LB-AMP was made by inoculation 1/100 with the pre-culture. This culture was grown at 30 °C while shaking until the optical density 600 nm reached 0.7. Protein expression was then induced by adding isopropyl β-D-1-thiogalactopyranoside (iPTG, Sigma-Aldrich, Cat # I6758) at a final concentration of 0.5 mM. The bacterial cells were harvested, washed and resuspended with the lysis buffer [50 mM Tris–HCl (Trizma® hydrochloride, Sigma-Aldrich, Cat # 857645) pH 8, 250 mM NaCl (Sigma-Aldrich, Cat # S9888), 30 mM imidazole (Sigma-Aldrich, Cat # I2399), 1 mg/ml Lysozyme (powder from chicken egg white, Sigma-Aldrich, Cat # L8876), antiproteases cOmplete™ EDTA free (Roche, Cat # 11873580001) and 1 U/ml Benzonase (Nuclease Benzonase® from Millipore, Sigma, Cat # E1014)] and incubated for 1 h at 4 °C. Lysis was completed by 6 cycles of 30 s of sonication. The disrupted cell lysate was centrifuged at 12,000 g during 20 min and the supernatant was loaded onto a 1 mL Ni_2_ + -NTA agarose column (Qiagen, Cat # 30210) preequilibrated with buffer A (50 mM Tris–HCl pH 8, 250 mM NaCl, 30 mM imidazole). The column was washed with 100 mL of buffer A. The protein was eluted by the buffer B (50 mM Tris–HCl, pH 8, 250 mM NaCl, 500 mM imidazole). The eluted sdAb (2C5) was further purified by high-resolution Superdex® S-75 10/300 gel filtration column (Cat # GE17-5174-01) with PBS, concentrated to 1 mg/mL and frozen at − 80 °C.

### In vitro characterization of the single-domain antibody

#### Enzyme-linked immunosorbent assay (ELISA)

Different antigens (native Tau, Tau oligomers, Tau fibers, β-amyloid fibers, AcR3 mimes, according to data obtained by AFM) were coated at 10 µg/mL final concentration onto 96-well plates overnight at 4 °C. Plates were then washed with PBS and the saturation of non-specific sites was achieved by depositing 200 μL/well of PBS, 0.05% Tween (Tween20: Sigma-Aldrich, Cat # P1379-500), 1% bovine serum albumin (BSA Fraction V, PanReac AppliChem, Cat # A1391.0250) for 1 h at RoT (room temperature). Then, increasing concentrations of 2C5 sdAb (0 to 10 μM diluted in PBS, 0.05% Tween, 1% BSA) were added to each well for 1 h at RoT, followed by 3-washed steps in PBS, 0.05% Tween. Then, the anti his-tag HRP (horseradish peroxidase) conjugated antibody (Miltenyi Biotec, Cat # 130-092-785, 1:5000, Mouse monoclonal Ab) was incubated as the secondary antibody for 1 h. Three-washed steps in PBS were followed by incubation with 100 µL 3,3',5,5'-Tetramethylbenzidine substrate (TMB, Sigma-Aldrich, Cat # T8665). The reaction was stopped by adding 100 μL 1N HCl (Sigma-Aldrich, Cat # H1758). Finally, the optical density at 620 nm was measured with a Varioscan Lux multiplate reader. The dissociation constant (Kd) was determined for each antigen in triplicate.

#### Immunohistochemistry (IHC) on Human brain tissue

Brains samples were obtained from the autopsy material collection from the Department of Mental Health and Psychiatry, University Hospitals and University of Geneva. The study included brain from authorized autopsies of well characterized neuropathological cases, including those of normal aging (respectively Braak stage I or II) and Alzheimer disease patients (Braak stage V).

Blocks of the hippocampus and adjacent entorhinal and temporal cortex were fixed by immersion in a solution of 4% paraformaldehyde (Sigma-Aldrich, Cat # HT501128) in PBS, pH 7.4, at 4  C for 7 days, and then included in paraffin (QPath, Cat # 10048501). IHC was conducted on 30-μm -thick adjacent paraffin embedded sections. The brain floating slices were first deparaffinized in successive xylene (1,4-dimethylbenzene, Sigma-Aldrich, Cat # 296333), ethanol (Honeywell, Cat # 32221), distilled water and PBS baths, then conserved in PBS, 0.01% sodium azide solution (NaN_3_, Sigma-Aldrich, Cat # 822335) until IHC. The slices were permeabilized in PBS, 0.2% Triton X100 (Sigma-Aldrich, Cat # T8787-500). The slices were unmasked with boiled citrate buffer (Antigen Unmasking Solution, Vector Laboratories, Cat # H-3300) pH 6, 3 times for 5 min. A step to block non-specific binding sites was carried out in TBS, 0.05% Tween (Tween20: Sigma-Aldrich, Cat # P1379-500), 1% BSA (BSA Fraction V, PanReac AppliChem, Cat # A1391.0250). The primary antibodies were applied overnight at 4 °C for the detection abnormal phosphorylated Tau with AT8 antibody (ThermoSc, Cat # MN1020, 1:600, Mouse monoclonal Ab), oligomeric form of Tau with T22 antibody (Merck, Cat # ABN454, 1:600, Rabbit polyclonal Ab), Aβ peptide with Aβ4G8 antibody (Biolegend, Cat # 800701, 1:800, Mouse monoclonal Ab), and with 2C5 sdAb (20 nM). The secondary antibodies (Vector laboratories, Cat # BA-9200, biotinylated Goat Anti-Mouse and Vector laboratories, Cat # BA-1000 biotinylated goat anti-rabbit) were applied for 1 h at RoT, except for 2C5 for which Anti-His-Tag antibody (Abcam, Cat # ab9108, 1:200, Rabbit polyclonal Ab) was applied for 24 h at 4 °C and tertiary goat anti-rabbit antibody (Vector laboratories, Cat # BA-1000), 1 h at RoT. The avidin–biotin complex (VECTASTAIN® Elite® ABC-HRP kit, Vector Laboratories, Cat # PK-6100) and 3,3'-diaminobenzidine (DAB Substrate, Peroxidase (HRP) with Nickel, Vector Laboratories, Cat # SK-4100) kits were successively used to amplified and reveal positive immunohistochemical reaction. Finally, the sections were placed on microscope slides, counterstained with hematoxylin (DiaPath, Cat # C0303), and mounted with coverslip and mounting medium (Vector Laboratories, Cat # H5700).

#### Thermal stability of 2C5 single-domain antibody

The thermal denaturation was monitored by circular dichroism (CD) using a Jasco (JASCO, Easton, MD) spectropolarimeter equipped with a Peltier temperature control system. Briefly, the samples (0.375 µg/µL) were prepared by dilution into ultra-pure water. CD spectra were obtained from 205 to 260 nm at temperatures ranging from 25 to 80 °C in a 10 mm quartz cuvette (band width of 4.0 nm with six accumulations of scans to determine the CD profile of the proteins). The changes in CD absorption as function of the temperature and at 215 nm were then compared.

### Radiolabeling and High-Performance Liquid Chromatography (HPLC) assessment of in vitro and in vivo stability

#### Radiolabeling

The 2C5 sdAb carrying a 6xHis Tag was labeled with Technetium-99m using the tricarbonyl method as described by Gainkam et al. [37]. Technetium-99m was selected for this study since it represents the most commonly used medical radioisotope. Indeed, thanks to its characteristics (half-life of 6 h and photon energy of 140 keV), it is perfectly adapted to nuclear imaging, specifically single-photon emission computed tomography (SPECT). First, ^99m^Tc-tricarbonyl, [^99m^Tc][Tc(CO)_3_(H_2_O)_3_]^+^, was synthetized by adding 1 mL (2.5–3 GBq) of freshly eluted sodium pertechnetate supplied by a ^99^Molybdenum / ^99m^Technetium generator (TEKCIS, Cis Bio) to a commercial kit (CRS Kit for tricarbonyl, Paul Scherrer Institut, PSI, Switzerland) containing 9 mg potassium sodium tartrate⋅tetrahydrate, 2.9 mg sodium tetraborate⋅decahydrate, 7.8 mg of sodium carbonate, and 4.5 mg disodium boranocarbonate. The vial was incubated at 100 °C in a boiling bath for 20 min, and after 10 min at RoT, the ^99m^Tc-tricarbonyl precursor was neutralized with HCl to pH 6.5–6.8. In the second step, 1 GBq of ^99m^Tc-tricarbonyl was added to 100 µg of 2C5 sdAb (10 MBq / µg) and then incubated at 50 °C for 90 min. The [^99m^Tc]Tc-2C5 solution was further gel filtrated on a NAP-5 column (Sephadex™ G-25 DNA Grade Gel, GE Healthcare) in PBS (Sigma-Aldrich, Cat # D8662) and filtered through a 0.22 µm filter (Millex, Millipore).

The quality control of the radiolabeled 2C5 was performed by radio-HPLC with a reversed-phase BioResolve™ column (2.7 µm, 150 × 2.1 mm, 450 Å, Waters). The mobile phase consisted of water-0.1% trifluoroacetic acid (TFA) (solvent A; Sigma-Aldrich, Cat # 302031) and acetonitrine-0.1% TFA (solvent B; ACN, Sigma-Aldrich, Cat # 34851) at a flow rate of 1 mL.min^−1^ using a gradient mobile phase: 0 to 5 min with 10% B; 5 to 12 min, of 10% to 90% B; the gradient remained at 900% B during 5 min before returning to the initial condition at 22 min. The radiochemical purity (RCP) of [^99m^Tc]Tc-2C5 was determined immediately after the radiolabeling and in the mixture of labeling at RoT to determine its stability over a period of 6 h. The percentage of the region of interest (%ROI) was used to determine the RCP. On each HPLC profile, all regions of interest were selected, i.e. every peak. The %ROI corresponds to the percentage of one selected region divided by the sum of all identified regions on the HPLC profile.

#### Determination of the partition coefficient (Log *P*)

The ability of the compounds to penetrate the BBB through passive diffusion could be due, at least in part, to their lipophilic nature. Indeed, lipophilic molecules can more easily pass through the cell membranes. The partition coefficient (log *P*) is a key parameter that determines the lipophilic nature of a compound. Positive log *P* values characterize lipophilic substances and negative log *P* values characterize hydrophilic ones. A higher log P (with a value ideally greater than 2), therefore a more lipophilic molecule, would be more suitable for crossing the BBB. Currently developed Tau tracers, presenting preclinical or clinical data confirming their passing through the BBB, have a log P between 1 and 4 (for example, 1.67 for [^18^F]F-AV-1451) [38].

The log *P* value was assessed by using liquid–liquid extraction followed by phase separation (n = 3). [^99m^Tc]Tc-2C5 (50 µL) was mixed in a 1:1 (v/v) mixture of n-octanol (500 µL, Sigma-Aldrich, Cat # 293245) and PBS (450 µL), followed by vigorous vortexing for 1 min. A clear separation of the two layers was obtained by centrifugation at 11,500*g* for 5 min. Both n-octanol phase and buffer phase were taken and counted separately using a dose calibrator (Capintec CRC-15R, Aries). This process was repeated by replacing fresh phosphate buffer and 1-octanol, respectively. Log *P* was calculated as the logarithm of the ratio of radioactivity measured in the n-octanol phase to the PBS buffer: Log *P* = log (total counts in n-octanol) / (total counts in PBS buffer).

#### In vitro stability studies

The in vitro stability of [^99m^Tc]Tc-2C5 was studied in mouse, rat and human blood (n = 3/condition). The radiotracer was incubated at final concentration of 37 MBq/mL in blood at 37 °C for 45 min. A sample of each mixture (100 µL) was withdrawn at 5, 10 and 45 min, and then centrifugated at 2000 g for 4 min to separate the plasma from the blood cells. A suitable volume of 10% trichloroacetic acid (TCA, Sigma-Aldrich, Cat # T6399) (0.1–0.12 µL / µL plasma) was added to plasma to precipitate proteins. After centrifugation at 11,500*g* for 3 min, the supernatant (protein‐free plasma fraction) was analyzed by radio-HPLC as previously described to assess the stability of the radiotracer.

#### In vivo stability study

The evaluation of [^99m^Tc]Tc-2C5 stability was determined in the mouse blood 5, 10 and 45 min after its intravenous injection (n = 3/time points). The animals were anesthetized using isoflurane and a transmural puncture was achieved to collect a blood sample directly from the left ventricular cavity. The protein‐free plasma fraction obtained as described above was analyzed by RP‐HPLC as described to evaluate the stability of radiotracer.

### In vivo evaluation

The distribution of the radiotracer was evaluated in 7-week-old females’ Swiss mice (Janvier Labs, France). [^99m^Tc]Tc-2C5 was injected intravenously into the mouse tail. The mice were then sacrificed by CO_2_ inhalation at 5-, 10- or 45-min post injection (4 mice per group). Samples of organs were harvested (gallbladder, brain, heart, stomach, liver, salivary glands, intestine, muscle, bone, pancreas, skin, lungs, spleen, kidneys, thyroid, bladder, blood), weighed, and the radioactivity was determined with a gamma counter (Wizard^2^, PerkinElmer). After correction of the radioactive decay, the data were analyzed to determine the percentage of the injected dose (ID) per gram of tissue (%ID/g).

All procedures were performed in accordance with the institutional guidelines and approved by the animal care and use committee of Grenoble University and the ad hoc French minister (n°02573.02).

### Statistical analysis

The results are presented as mean ± standard errors. The Mann–Whitney test was used for biodistribution study to compare unpaired datasets with unequal variance between 5- and 45-min post injection. Kruskal–Wallis non-parametric one-way ANOVA was used to compare RCP at different time point in comparison to the radiolabeling. Differences were considered significant at a p level of less than 0.05. The analyses were performed using GraphPad Prism 9.0.

## Results

### 2C5 sdAb production

**Fig. 1:**
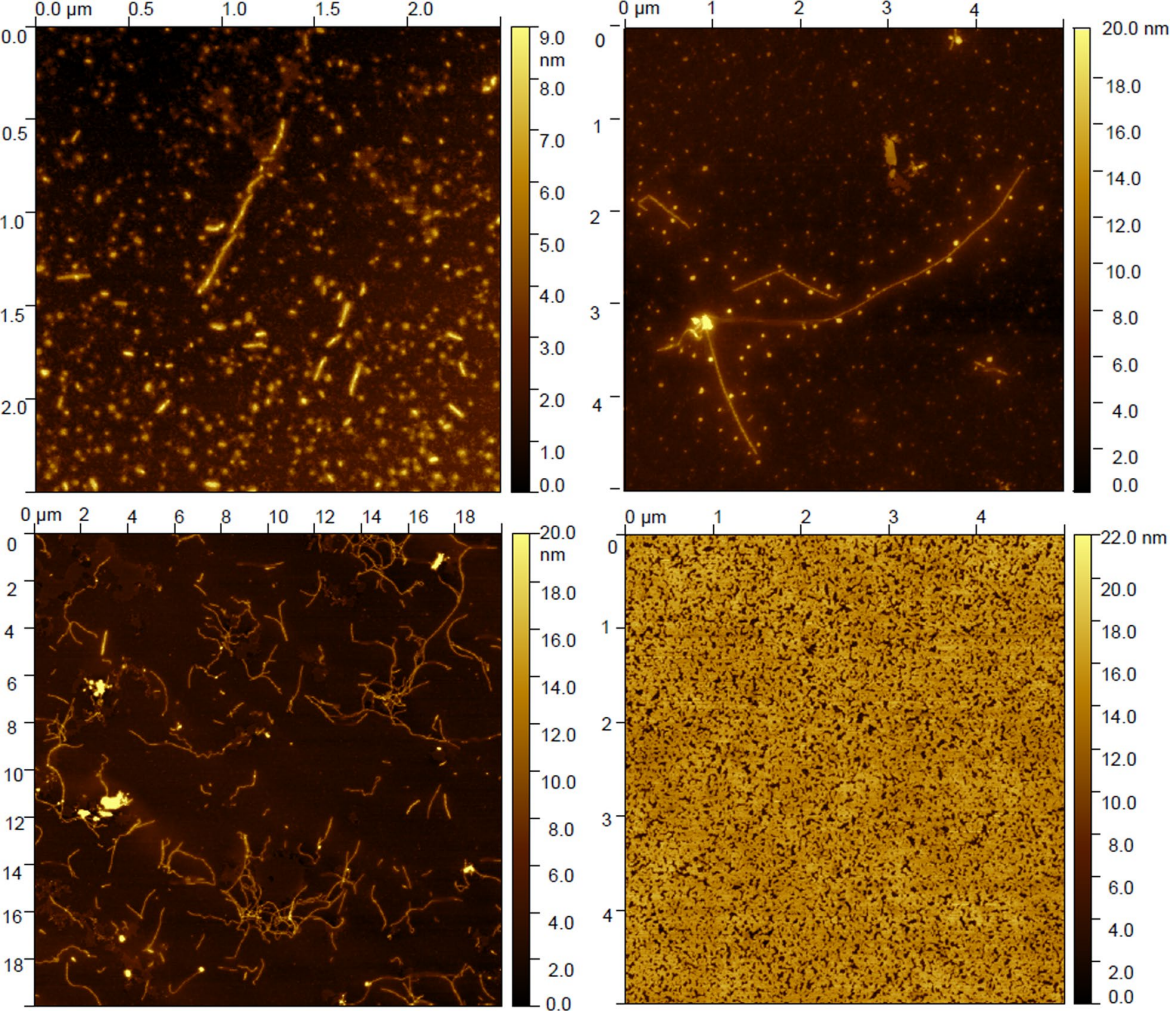
Atomic force microscopy images of Tau and AcR3 species depending on the aggregation time. After 48 h of incubation in the aggregation medium, Tau protein mainly forms oligomers with only a few short fibers (**A**). At 72 h, longer fibers are observed but oligomer species remain abundant (**B**), and at 16 days, Tau protein is mainly aggregated in fibers (**C**). As for AcR3 peptide, fibers covering the entire area are already formed within 24h (**D**)

Prior to llama immunization, the oligomers and fibers obtained in vitro from the native htau40 protein and AcR3 peptide were characterized by AFM (Fig. 1). After 48 h of aggregation in the presence of heparin, AFM showed that the Tau protein mainly formed oligomers with only a few short fibers present (Fig. 1A), while after 72 h of aggregation longer fibers were observed associated with the oligomers (Fig. 1B). After 16 days of aggregation, the Tau protein was mainly found as long fibers (Fig. 1C). For the AcR3 peptide, abundant fibers covering the entire area were observed already at 24 h (Fig. 1D).

After the llama immunization, multiple clones were isolated and subsequently purified. A phage display selection and a fast screening in ELISA assay of the resulting sdAb library led to the isolation of the 2C5 sdAb. The different antigens were challenged, including Tau oligomers (aggregation stopped at 48 h, Tau-O), Tau fibers (aggregation until 72 h, Tau-F), AcR3 as Tau mimes (at 24 h) according to AFM data, as well as Tau native protein (Tau-N) and Aβ fibers (Aβ-F). The 2C5 sdAb showed the highest selectivity for Tau oligomers *versus* native Tau protein and Aβ fibers. The 2C5 sdAb is composed of 143 amino acids, resulting in a molecular weight of 15492 Da, and a theoretical isoelectric point of 7.18. This sdAb contains at the C-terminal end a Myc Tag, a poly-histidine Tag and a GS tag.

### In vitro characterization

#### ELISA assays

After production in *E. Coli*, to confirm the high selectivity and specificity of the 2C5 sdAb and to estimate apparent affinities, a complete ELISA assay was performed with the different antigens.

**Fig. 2:**
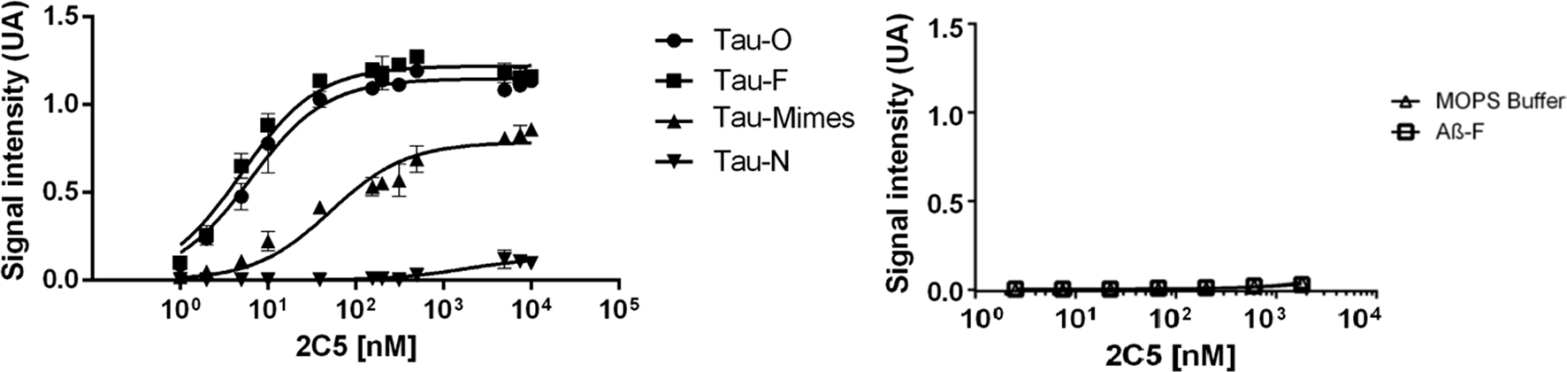
ELISA assays for 2C5 single-domain antibody with different species. The ELISA assays for 2C5 sdAb with Tau oligomers at 48 h (Tau-O), Tau fibers at 72 h (Tau-F), Tau mimes at 24 h (the peptide covering the R3 region of the repeated motifs of Tau) and native Tau (Tau-N), and with Aβ fibers (Aβ-F). Values are represented with standard error for triplicate

The 2C5 apparent affinity was high for the Tau oligomers (Kd = 6.3 ± 0.6 nM) and for the fibrillar form of Tau (Kd = 5.0 ± 0.5 nM), as well as for AcR3 aggregates as Tau mimes (Kd = 51.7 ± 8.7 nM) (Fig. 2, left panel). Its apparent affinity was however quite low for the native Tau protein (Kd = 1791 ± 735 nM). Furthermore, the specificity was also verified, revealing a minimal binding to β-amyloid peptide (Kd > 10,000 nM) (Fig. 2, right panel).

#### Immunohistochemistry

**Fig. 3:**
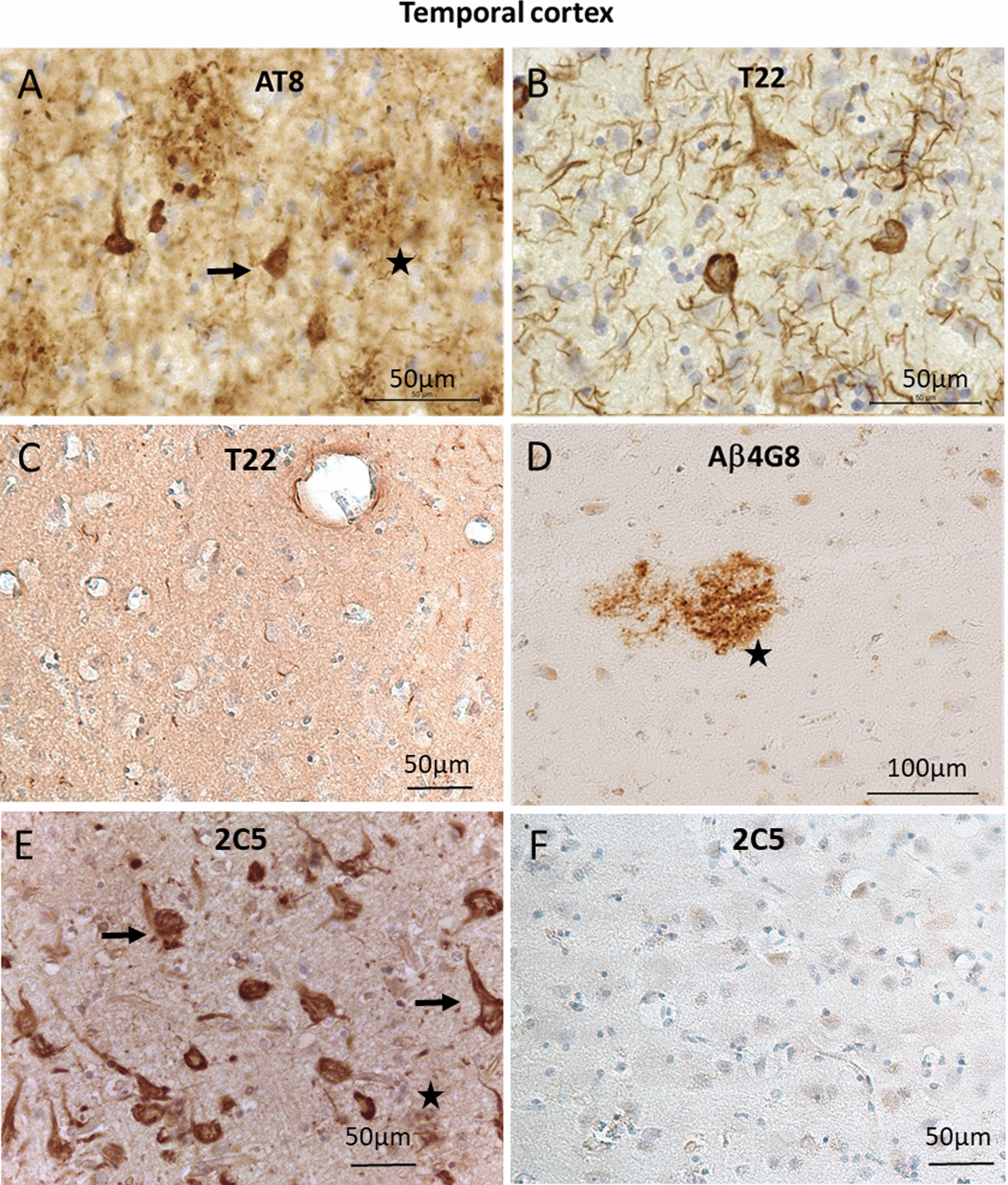
Immunohistochemistry with 2C5 sdAb and reference antibodies on human brain slices from AD and control patients. Immunohistochemistry was performed with the following reference antibodies: Tau AT8 (**A**), Tau T22 (**B**, **C**), and amyloid Aβ 4G8 (**D**) in the temporal cortex of a AD Braak V subject (**A**, **B**, **D**) and control Braak I (**C**). (**E**) Immunohistochemistry in the AD (Braak V) subject with the 2C5 sdAb, revealing staining that resembles Tau AT8, Tau T22 and 4G8 antibodies. (**F**) Immunohistochemistry in the temporal cortex of a control subject with the 2C5 sdAb, revealing low intracellular staining. Black arrows identify neurites and neuron cell bodies, black stars localise extracellular deposits. Slices were hematoxylin counterstained. Scale bars were indicated in each image

**Fig. 4:**
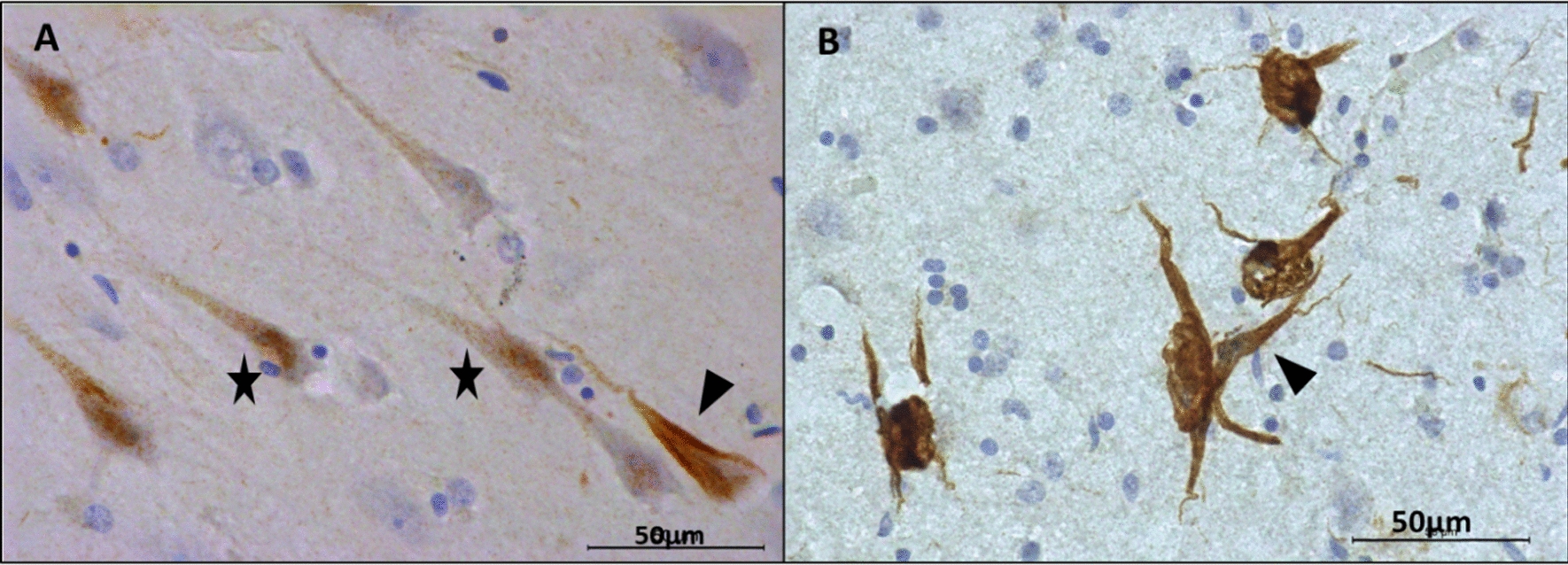
Anatomopathological analysis of the brain slices from AD patients showing several types of 2C5 staining. At the early stages of the disease, one can observe a punctiform labeling (indicated by black stars), due to the presence of Tau oligomers, which initially appeared at the level of the axonal extensions, then to the cell bodies of the affected neurons (**A**) in the entorhinal cortex, and (**B**) in the hippocampus. The fibrillar forms (black arrowhead) of the Tau protein were detected in the cell bodies of neurons affected at the more advanced stages. For the scale reference, the black line in the right lower corner represents 50 µm

To identify the specificity of 2C5 sdAb labeling, an immunohistochemical analysis was performed on the brain of AD subjects including a representative subject (female patient, 81 years old, Braak V). Detection of AD Tau forms was performed with two reference antibodies, AT8 and T22, identifying poly-phosphorylated and oligomeric forms of Tau, respectively. AT8-positive star-shaped neurons were detected in the entorhinal cortex (not shown) and temporal (Fig. 3A). Extracellular labeling was also observed, corresponding to neuritic plaques (Fig. 3A). Oligomeric forms of Tau were visualized in both neurites and neuronal cell bodies (Fig. 3B) in contrast to the absence of any staining in the temporal cortex of control subject (male patient, 70 years old, Braak I) (Fig. 3C). The presence of amyloid plaques was revealed by the Aβ4G8 antibody (Fig. 3D). Labeling with 2C5 sdAb on sections from the same subject revealed labeling of neurons and their neurites as well as extracellular clusters, resembling neuritic plaques (Fig. 3E). The absence of extracellular labeling and the very low intracellular neuronal labeling in the control subject confirmed the specificity of 2C5 sdAb staining (Fig. 3F). In the control subject, only rare neurons were similarly detected in the transentorhinal cortex without labeling observed in the temporal cortex or neocortical areas. In the AD subject, at higher magnification, punctiform 2C5 sdAb staining attributed to oligomers in the entorhinal cortex and fibrillar forms of the Tau protein in neurons affected by advanced stages of the Tau disease in the hippocampus were observed (Fig. 4).

### Radiolabeling and stability

**Fig. 5:**
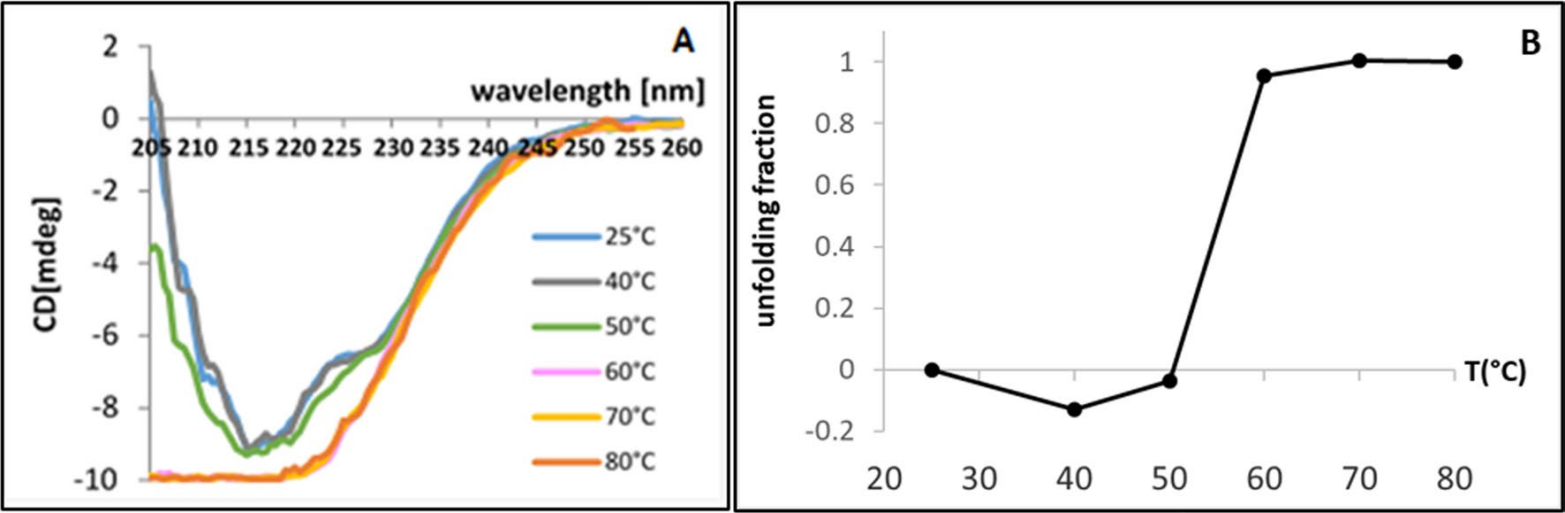
Circular dichroism of 2C5 sdAb. (**A**) Circular dichroism spectra of 2C5 sdAb from 25 °C to 80 °C. (**B**) Unfolding fraction as function of the temperature and at 215.5 nm

The duration of the radiolabeling procedure can be reduced by increasing the incubation temperature of the ligand with the radioelement. Before the radiolabeling assay, thermal unfolding of 2C5 sdAb was then monitored by CD at different temperatures, from 25 to 80 °C (Fig. 5A), to determine the maximal temperature which can be used without affecting the secondary structure of the protein. Considering the results obtained showing that at 60, 70 and 80 °C 2C5 sdAb was unfolded (Fig. 5B), 50 °C was chosen as the best heating temperature compromise.

**Fig. 6:**
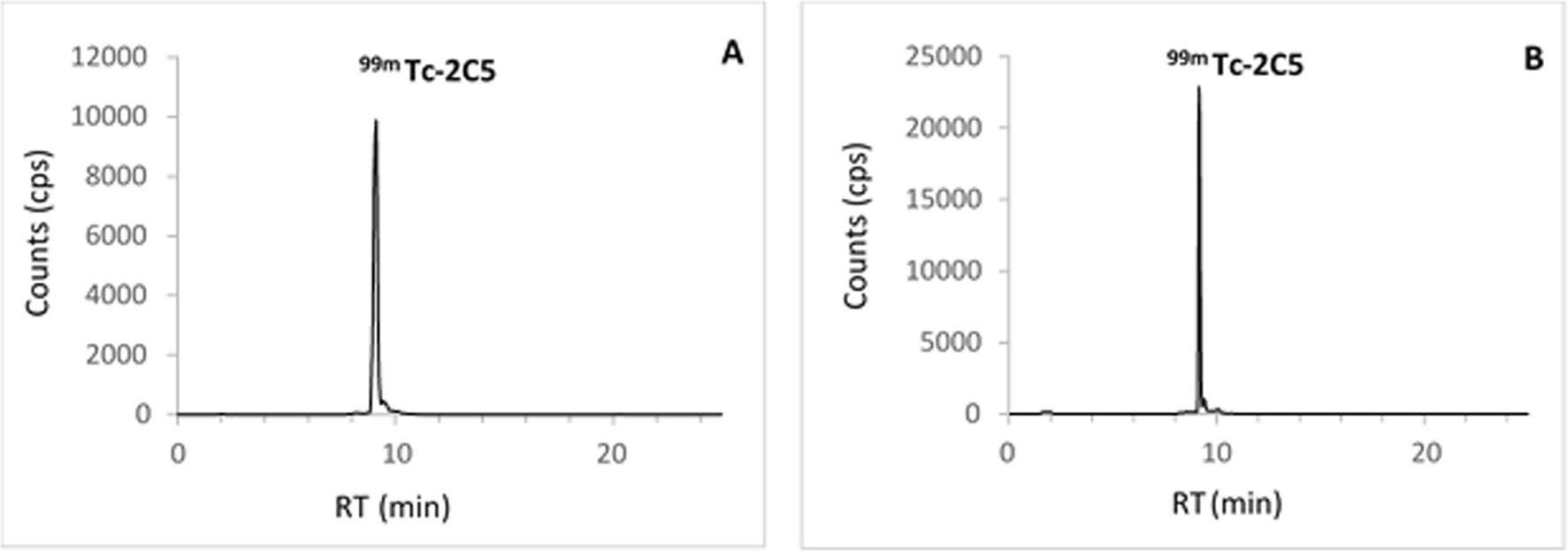
Representative radio-HPLC profiles of [^99m^Tc]Tc-2C5. Quality controls performed (**A**) immediately following radiolabeling, and (**B**) 6 h after. RT: retention time, in minutes

2C5 sdAb was successfully radiolabeled with ^99m^Tc (n = 4), with an average retention time (RT) and RCP of 9.1 ± 0.03 min and 95.0 ± 0.4% respectively, immediately after radiolabeling and purification. A representative profile is provided in Fig. 6A. [^99m^Tc]Tc-2C5 remained stable at 6 h after radiolabeling with an average RT and RCP of 9.18 ± 0.07 min and 94.6 ± 1.2% respectively. A representative profile is provided in Fig. 6B.

The Log *P* value of [^99m^Tc]Tc-2C5 was found at -2.37 ± 0.09 suggesting rather its hydrophilic character.

**Fig. 7:**
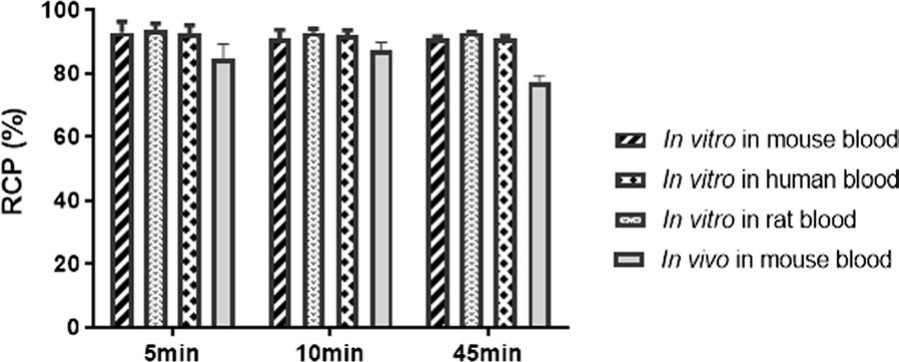
In vitro and in vivo stability of [^99m^Tc]Tc-2C5. The stability of [^99m^Tc]Tc-2C5 was assessed (**A**) in vitro after 5, 10 and 45 min incubation in the mouse, rat and human blood, and (**B**) in vivo at 5, 10 and 45 min after intravenous injection in mouse. RCP (radiochemical purity) is expressed as a percentage of the mean value of triplicate at each time and standard deviation

Stability studies in vitro achieved in mouse, rat and human blood showed similar patterns over time (Fig. 7). At the time points of 5, 10 and 45 min, the RCP was of 93.0 ± 3.4, 90.9 ± 2.8 and 91.1 ± 0.6% after incubation in mouse blood (p > 0.05), 95.0 ± 2.0, 93.6 ± 1.1 and 92.8 ± 0.2% after incubation in rat blood (p > 0.05), 92.5 ± 2.8, 92.0 ± 1.6 and 91.6 ± 0.7% after incubation in human blood (p > 0.05), respectively. According to these results, after 45 min incubation in the blood in vitro, [^99m^Tc]Tc-2C5 remained highly stable with a RCP higher than 90%.

In vivo, the stability was also studied at 5, 10 and 45 min after intravenous injection in mice. The RCP of [^99m^Tc]Tc-2C5 was of 84.5 ± 4.8 (p > 0.05), 87.1 ± 2.6 (p > 0.05), 77.2 ± 2.2% (p < 0.01) at 5, 10 and 45 min, respectively, compared to the RCP at the injection time. One peak appeared at a RT of 8.6–8.7 min and represented of 2 to 11%, most likely corresponding to a metabolite while merely no free ^99m^Tc was observed.

### In vivo evaluation

**Fig. 8:**
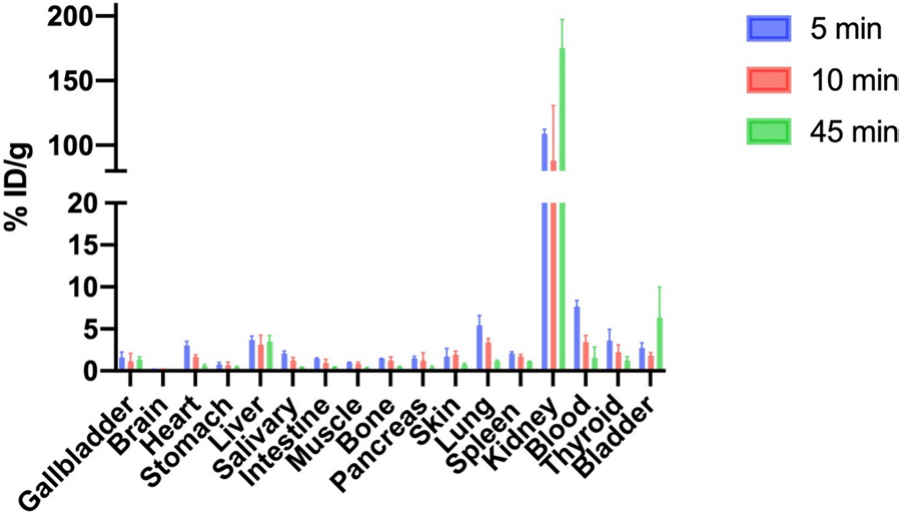
Biodistribution of [^99m^Tc]Tc-2C5 in healthy mice. Biodistribution at 5, 10, and 45 min post injection (n = 4 per group). Data are expressed as a percentage of the injected dose taken up per gram of tissue (% ID/g). Mean values ± SD are represented. Salivary represents the salivary glands

To assess the ability of [^99m^Tc]Tc-2C5 to target the brain in vivo and to describe the main organs capturing the single-domain antibody, biodistribution in healthy mouse was performed (Fig. 8). The mice were injected with a mean activity of approximately 31 MBq. [^99m^Tc]Tc-2C5 was rapidly cleared from the circulation, with a blood activity of 7.64 ± 0.72% ID/g at 5 min post injection, decreasing at 3.41 ± 0.80 and 1.52 ± 1.32% ID/g at 10 and 45 min (p < 0.05), respectively. The blood activity was mainly contained in plasma, rather than being associated with red blood cells. Uptake in the kidneys was largely predominant, representing 108.85 ± 3.47, 87.84 ± 42.79 and 174.96 ± 22.45% ID/g at 5, 10 and 45 min respectively (p < 0.05), corresponding to the main elimination route. Expect for the lungs at 5 min post-injection, uptakes were lower than 4% ID/g in all the other investigated tissues. More precisely, we observed [^99m^Tc]Tc-2C5 activities of 1.63 ± 0.57% ID/g in the thyroid and 0.61 ± 0.34% ID/g in the stomach at 45 min, testifying to the low presence of free ^99m^Tc. The radioactivity in the brain was measured at 0.17 ± 0.03, 0.12 ± 0.07 and 0.02 ± 0.01% ID/g at 5, 10, and 45 min post-injection respectively (p < 0.05), following the decrease in blood radioactivity and demonstrating no uptake of the radiotracer.

## Discussion

The research for AD biomarkers has significantly improved its differential diagnosis [39]. However, there are still great clinical diagnosis uncertainties, especially during the early asymptomatic preclinical phase of AD. We are gradually moving towards a biological definition of AD such as in the ATN classification requiring biomarkers for β-amyloid lesions, Tau lesions and neurodegeneration [40]. Tau protein aggregates, forming intracellular insoluble NFTs, appear to be involved in the early stage of AD neurodegenerative processes [41]. Indeed, NFTs are present in the entorhinal-hippocampal structures before Aβ plaques in the cortex, and the cortical NFTs distribution and number correlate with the memory impairments of AD patients. More than the mature intracellular NFTs, Tau oligomers described in AD, in the neuropil and in the perivascular spaces of cerebral vessels, are currently regarded as the toxic Tau species responsible for the spread of the disease from one neuron to another with a prion-like fashion propagation [20, 42–44]. These toxic Tau species lead to the dramatic impairment of the synaptic function and neurodegeneration.

Moreover, different promising active and passive immunotherapeutic strategies directed against the pathological forms of Tau, and whose impact onto Tau oligomers was demonstrated in preclinical protocols, are currently undergoing clinical trials [14–16]. The development of such therapeutic strategies requires the detection and follow-up of the early neurodegenerative signs of the disease, especially the Tau oligomers presence, in the population at risk of AD to select potential responsive patients and to monitor treatment effects. An appropriate AD biomarker will greatly reduce costs associated with drug development by enabling selection of a more homogeneous patient population and evaluation.

Our research aims to provide a single-domain antibody, named 2C5, as a new radiotracer to efficiently detect and monitor oligomeric forms of Tau in human brain. We selected one camelid sdAb, named 2C5, from a screening of clones isolated from a llama immunized with the largest human Tau isoform hTau40 and R3 mimes at different maturation states (oligomers and fibers). Before radiolabeling, 2C5 sdAb appears to be a relevant tool for detecting the early forms of Tau pathology. Especially, the 2C5 sdAb is able to recognize the soluble Tau oligomers with nanomolar affinity and a good specificity, with in particular an absence of affinity for the native Tau protein and the Aβ protein. This sdAb allows immuno-histological labeling of pathological forms of Tau in AD brain tissues with a good contrast, very similarly to that obtained with AT8, the commercialized Tau phosphorylated antibody, considered as the gold standard method recognizing the Tau protein phosphorylation of Ser 202 and Thr 205, and superimposable to the lesions revealed by T22, a commercial polyclonal antibody described as recognizing Tau oligomers (Tau-441(2N4R)) [42]. More precisely, it is possible to highlight with 2C5 sdAb a punctiform labeling of the Tau oligomers in the neurons at an early stage of the disease, as well as the fibrillar forms in more advanced AD stages.

Some lesions in the form of plaques were identified in the different temporal cortical sites, including in the trans entorhinal cortex, with 2C5 sdAb but also with the reference antibodies. They probably correspond to plaques containing phosphorylated Tau protein and resulting from the interactions between Aβ and Tau fragments [45]. However, these particular lesions seemed to be later lesions than the marked neurons.

The in vivo biodistribution study of [^99m^Tc]Tc-2C5 was performed in healthy mice. Initially, 2C5 sdAb was successfully radiolabeled with technetium-99m using the well-known tricarbonyl method, with a high radiochemical purity and showing a high stability. As expected because of their small size, 2C5 sdAb exhibited a rapid clearance from the bloodstream in vivo*,* primarily through renal excretion. Unfortunately, these studies did not reveal any significant activity in the cerebral parenchyma.

Nuclear imaging with SPECT or PET tracers has the potential to play a crucial role in the early and differential diagnosis of AD, in the follow up of longitudinal progression and to support the development of novel therapeutic approaches, by visualizing the Tau deposits in vivo, by providing information on the regional distribution and the spatiotemporal evolution of the disease [46].

Thereby, Tau imaging has made great progress in the past few years with many Tau PET tracers that have been developed so far [47]. First, small molecules targeting β-sheet structures have already been developed for PET imaging of NFTs. The most studied tracer ^18^F-Flortaucipir (also known as ^18^F-AV-1451 or T807) [24] has been approved by the US Food and Drug Administration in 2021 to estimate the density and distribution of aggregated Tau NFTs in patients being evaluated for AD [25]. The first-generation of Tau PET tracers also includes tracers from the THK family and ^11^C-PBB3. These radiotracers allowed to correctly detect and quantify Tau deposition in the brain at the advanced AD Braak stages (> stage IV) making it an accurate diagnostic and prognostic biomarker [48]. One of the main issues for these radiotracers is that β-sheet structures do not only appear in misfolded Tau, but also in other proteins such as Aβ peptides leading to the significative off-target binding including the choroid plexus, basal ganglia and substancia nigra [49]. The off-target binding profile varies widely across Tau PET tracers. Second generation of Tau PET tracers have thus been developed, such as ^18^F-RO948 [50, 51] or ^18^F-MK6240 [52, 53], to significantly lower off-target binding and obtain high specific NFTs binding. However, despite the considerable progress in this research for NFTs brain PET ligands and faced with the need for an early diagnosis approach, we notice that no tracer is currently available to detect toxic oligomeric forms of Tau. AD being a worldwide disease, we initially chose to radiolabel our sdAb with ^99m^Tc with the idea of obtaining a tracer that can be used anywhere in the world in SPECT imaging.

SdAbs exhibit relevant features for target recognition and imaging agent development. Some authors have already identified sdAb recognizing different forms of the Tau protein. SdAb that labeled NFTs have been reported using phosphorylated Tau enriched AD brain extracts as antigens [54]. Dupré et al. [55] demonstrated the ability of F8-2-derived sdAbs to specifically bind their Tau target in transgenic mouse brain tissues, in the same manner as an entire IgG. Danis et al. [56] modified the biochemical properties of their Z70 single-domain antibody to bind the Tau microtubule-binding domain in the intracellular compartment. In our team, we developed the 2C5 sdAb to specifically target the Tau oligomers as an earlier pathological signature of AD, with good in vitro labeling using ELISA assay but also on human brain sections from AD patients. While 2C5 sdAb also recognizes the Tau fibers, it remains of great interest, the oligomers being the hallmarks of the earlier stage of the pathology.

The main limitation in the further development of this sdAb as a radiotracer targeting the Tau oligomers is its sharply insufficient BBB crossing observed during the biodistribution study in healthy mice. The barrier properties of the BBB [57] are stringent enough to restrict > 98% of small molecules and passive diffusion of all large molecules. Certain large or polar molecules can cross the healthy BBB, although this is tightly regulated by specific receptor/transporter-mediated processes. For example, it has been shown that < 0.1% of conventional immunoglobulins can penetrate into the brain parenchyma. In our study, the use of healthy mice devoid of pathological Tau lesions may partly explain the absence of significant distribution of the radiotracer in the brain during in vivo studies. Therefore, it is crucial to identify a relevant animal model that presents human Tau oligomers to further develop this radiotracer.

Theoretically, sdAb are more suitable tools than antibodies to cross the BBB, mostly because of their lower molecular weight, and because lacking the Fc Fragment, they cannot be exported from the brain via the Fc receptor mediated efflux system. Therefore, some sdAbs have been reported to naturally cross the BBB. However, the basic isoelectric point seems to be a common criterion for sdAb having a significant passage of the BBB [54, 58, 59]. Following intravenous administration in transgenic mice and using two photon microscopy, Li et al.[60] demonstrated that the sdAb R3VQ (IP = 7.5) and A2 (IP > 9.5) significantly cross the BBB and specifically recognize their target, respectively extracellular β-amyloid and intracellular NFTs. Caljon et al. [61] demonstrated that sdAbs, using sdAb_An33 as model molecule, were able to cross the BBB at a detectable level but with only a very small proportion (0.0005% ID), while a significant increase was noted in an inflammation model with alterations of the BBB in late encephalitic stage animals. The authors highlighted the role of the rapid blood clearance in the poor CNS penetration of the sdAbs. The Fc5 and Fc44 sdAbs [62] transmigrate across an in vitro model of BBB but also after intravenous injection (until 4.5 ± 2.7% ID/g for Fc44) confirming the possibility for some sdAbs to naturally cross the BBB. The authors even evoked the possibility to use these sdAbs as carrier-vector to facilitate the passage of other molecules. The isoelectric point of 2C5 sdAb is limited (IP = 7.18), but above all, this single-domain antibody appears very little lipophilic which probably contributes to its low spontaneous passage. Especially, the Log *P* value of [^99m^Tc]Tc-2C5 was found clearly negative at − 2.37 ± 0.09 suggesting rather its hydrophilic character while tracers known to reach the brain, including the Tau PET tracers already developed, have a positive Log *P* and generally greater than 2.

There are several traditional methods for increasing the drug delivery into the brain that can be applied to sdAb [63]. In order to enhance the capability of 2C5 sdAb to cross the BBB and to increase its brain availability, protein engineering could be performed to appropriately modify its sequence to yield improved brain penetrating properties without losing the beneficial size and affinity properties. The first mechanism to target is the adsorptive transcytosis, which requires an electrostatic interaction, by means of the increase of positive charges of the molecule to interact with the negative charges at the endothelial cells surface. For this purpose, we will select different Cell-Penetrating Peptides (such as TAT, polyArginine) that could be fused to the sdAb. The second classic mechanism allowing the BBB crossing is the receptor mediated transcytosis, in this case the idea is to use a transmembrane receptor (classically transferrin receptor or insulin receptor) at the surface of the endothelial cells. This mechanism was employed by Wouters et al. [58] who developed an anti-transferrin receptor sdAb that can reach the brain via the receptor-mediated transcytosis after peripheral administration. Alternatively, among the most described methods, focused ultrasound (FUS) coupled with the infusion of microbubbles has been studied in recent years and is regarded as a noninvasive approach to disrupt the BBB in a transient and reversible manner, efficient and well tolerated in animals but also in the first human clinical trials [64, 65].

## Conclusions

The development of new biomarkers for the early and specific diagnosis of Alzheimer’s disease and its longitudinal follow-up appears crucial for a better patient care. The 2C5 sdAb is characterized by an excellent affinity and specificity for the Tau oligomers. However, the absence of a significant passage through the BBB requires modifications before being able to consider its use in nuclear medicine imaging in humans.

## Data Availability

The datasets analyzed during the current study are available from the corresponding author on reasonable request.

## Abbreviations

AD: Alzheimer’s disease
Aβ: Amyloid β peptide
NTF: Neurofibrillary tangles
PET: Positron emission tomography
SdAb(s): Single-domain antibody(ies)
BBB: Blood brain barrier
MOPS: 4-Morpholinopropane sulfonic acid buffer
ACR3: Acetylated peptide covering the R3 Tau region
PBS: Phosphate buffered saline
BSA: Bovine serum albumin
AFM: Atomic force microscopy
LB-AMP: Lysogeny Broth medium with ampicillin
ELISA: Enzyme-linked immunosorbent assay
RoT: Room temperature
Kd: Dissociation constant
IHC: Immunohistochemistry
CD: Circular dichroism
RT: Retention time
HPLC: High-performance liquid chromatography
SPECT: Single-photon emission computed tomography
RCP: Radiochemical purity
ID: Injected dose
